# 920 Get with the Times: Change is Hard but Bad Practice is Harmful!

**DOI:** 10.1093/jbcr/iraf019.451

**Published:** 2025-04-01

**Authors:** Kimberley Benzick

**Affiliations:** University of Texas Southwestern Parkland Burn Center

## Abstract

**Introduction:**

Change is HARD! People talking about the good ol’ days before smartphone and social media. But times have changed! The new generation has grown up with electronics. They have been able to access all information from the Web within seconds. So why do we struggle in hospitals to change to a virtual format for our education & learning materials? We know there is more up-to-date information, yet it takes time, money, and effort to swap out old references for new, or even to find where all that old bad information is hidden! BUT there is a solution... Virtual binders provide up-to-date information and education at the staff’s fingertips accessible from anywhere securely.

**Methods:**

The Nurse Educator created a virtual binder to house current information on current topics and new initiatives.

All staff were taught how to use the platform, access from their phone, workstation or any electronic device. All new information are added upon training and staff can utilize an anonymous virtual suggestion box to report training needs. All staff have access to all topics. Not only does this increase knowledge base for other units, but it also promotes collaboration and teamwork with the other disciplines through recorded webinars provided by from hospital wide leaders and providers.

**Results:**

Staff was initially resistant to change. However, over time they have reported a preference for this form of educational messaging. They state that having all materials in one location has eased the burden of attempting to locate paper copies as well as ensure that the paper documents were current best practice. Ease of use and portability of information have been of highest interest. New nurses report satisfaction with the ability to review information at their leisure. Seasoned nurses state that having quick set up guides for less common procedures is helpful to have on demand. Some nurses have reported reviewing learning topics or listening to recorded lectures while commuting. Nurses have reported increased knowledge of current standards of practices.

**Conclusions:**

The change from paper books and paper binders to the virtual binder was an initiative that was worthwhile! While change is hard, ill-informed or bad practices have the potential to harm patients. This method has provided staff with current resources at their fingertips on any platform. This has increased staff’s knowledge base and ensured current content being circulated through the unit, decreasing time for circulation of new materials, decreasing spending on printing and allowed for quick updates by the editor as new information and practices are implemented.

**Applicability of Research to Practice:**

Since the implementation of the virtual binder in the Burn Unit, this author has since facilitated the use of virtual binders for various other areas and staff levels in the facility. This is an initiative that can be implemented easily in other facilities.

**Funding for the Study:**

N/A

